# *De novo* characterization of the *Baphicacanthus cusia*(Nees) Bremek transcriptome and analysis of candidate genes involved in indican biosynthesis and metabolism

**DOI:** 10.1371/journal.pone.0199788

**Published:** 2018-07-05

**Authors:** Wenjin Lin, Wei Huang, Shuju Ning, Xiaohua Wang, Qi Ye, Daozhi Wei

**Affiliations:** 1 School of Life science, Fujian Agriculture and Forestry University, Fuzhou, China; 2 Fujian Key Laboratory of Medical Measurement, Fujian Academy of Medical Sciences, Fuzhou, China; 3 School of Crop Science, Fujian Agriculture and Forestry University, Fuzhou, China; Youngstown State University, UNITED STATES

## Abstract

*Baphicacanthus cusia* (Nees) Bremek is an herb widely used for the clinical treatment of colds, fever, and influenza in Traditional Chinese Medicine. The roots, stems and leaves can be used as natural medicine, in which indigo and indirubin are two main active ingredients. In this study, quantification of indigo, indirubin, indican and adenosine among various tissues of *B*. *cusia* was conducted using HPLC-DAD. Leaves have significantly higher contents than stems and roots (380.66, 315.15, 20,978.26, 4323.15 μg/g in leaves, 306.36, 71.71, 3,056.78, 139.45 μg/g in stems, and 9.31, 7.82, 170.45, 197.48 μg/g in roots, respectively). *De novo* transcriptome sequencing of *B*. *cusia* was performed for the first time. The sequencing yielded 137,216,248, 122,837,394 and 140,240,688 clean reads from leaves, stems and roots respectively, which were assembled into 51,381 unique sequences. A total of 33,317 unigenes could be annotated using the databases of Nr, Swiss-Prot, KEGG and KOG. These analyses provided a detailed view of the enzymes involved in indican backbone biosynthesis, such as cytochrome P450, UDP-glycosyltransferase, glucosidase and tryptophan synthase. Analysis results showed that tryptophan synthase was the candidate gene involved in the tissue-specific biosynthesis of indican. We also detected sixteen types of simple sequence repeats in RNA-Seq data for use in future molecular mark assisted breeding studies. The results will be helpful in further analysis of *B*. *cusia* functional genomics, especially in increasing biosynthesis of indican through biotechnological approaches and metabolic regulation.

## Introduction

*Baphicacanthus cusia* (Nees) Bremek (*B*. *cusia*) also named *Strobilanthes cusia* is a perennial medicinal herb distributed broadly in Fujian, Zhejiang, Guangdong, Hainan, Guangxi, Hong Kong, Taiwan and other southern areas of China. It is also distributed across Bangladesh, India, and Myanmar, from the Himalayas to Indo-China [1]. It has been cultivated in Fujian Province for more than 1000 years since the Song Dynasty. Indigo Naturalis is the leaf and stem extract of *B*. *cusia*, whose commercial name is “Qingdai” [2], and Baphicacanthus cusiae Rhizome et Radix is derived from its root [3], with the commercial name of “Nanbanlangen”. Previous studies have shown that Qingdai as an active ingredient was used to treat dental ulcers [4], ulcerative colitis [5–7], and psoriasis [8–10], and that Nanbanlangen has amany pharmacological applications, including antibacterial [11], antivirus [12], and anti-inflammatory effects [13, 14].

Chemical research has indicated that Qingdai and Nanbanlangen both contain many active ingredients, such as indigo [15] and indirubin [16]. Indican has been known to be a precursor of indigo and indirubin [17–19], the content of which was different in the leaf and root of *B*. *cusia* [15, 20]. To better understand the biosynthesis of indigo, gene expression possibly involved in indigo biosynthesis have been analysed by RNA-Seq in *Indigofera tinctoria* and *Polygonum tinctorium* [21], species closely related to *B*. *cusia*. However, these organisms belong to different genera and species classifications and differ in morphological traits.

The Qingdai produced in Fujian Province of China, named Jianqingdai, is a well-known genuine regional drug in China because of its good quality, but its effective constituent biosynthesis mechanism *in vivo* is still unclear. Until now, only nine proteins have been identified in the *B*. *cusia* leaf [22, 23], and 98 nucleotide sequences are available for *Baphicacanthus cusia*, but none of the nucleotide sequences of *B*. *cusia* can be found in NCBI GenBank, it is hard to clarify its molecular mechanisms due to the lack of information of genomic sequences, especially sequences of indican biosynthesis and metabolism genes. Therefore, our primary work is to obtain and characterize the transcriptome of *B*. *cusia*.

RNA-Seq next-generation sequencing (NGS) has been widely used in the analysis of transcriptomes in crops or vegetables, such as rice [24], barley [25], *Sorghum bicolor* [26], *Withania somnifera* [27], *Brassica rapa* [28] and Chinese cherry [29]. It has also been used to illustrate active components of biosynthetic and metabolic pathways in traditional Chinese medicine or identify differentially expressed genes (DEGs) between distinct sample groups such as *Isatis indigotica* [30–34], *Salvia miltiorrhiza* [35, 36], *Taxillus chinensis* [37], *Lonicera japonica* [38], *Toona sinensis Roem* [39], *Pseudostellariae radix* [40], *Eucommia ulmoides Oliver* [41], *Cistanche deserticola* [42], *Lycium chinense Mill* [43] and *Gentiana macrophylla* [44].

In this study, we measured the amounts of indigo, indirubin, adenosine and indican in the leaves, stems and roots of *B*. *cusia*. To understand the biosynthesis and metabolism pathways of indican in root, stem and leaf tissues of *B*. *cusia*, we generated multi-tissue transcriptomic data of *B*. *cusia* using RNA-Seq and annotated the multi-tissue transcriptome using publicly available databases and tools; we then ascertained genes that may be related to indican biosynthesis and metabolism pathways. Here, we report simple sequence repeats (SSR) in leaves, stems and roots from *B*. *cusia* and a number of differentially expressed genes (DEGs), particularly, cytochrome P450 (CYP450), UDP-glycosyltransferase (UGT), glucosidase and tryptophan synthase, that may be related to differential expression in indican biosynthesis and metabolism among leaves, stems and roots. Based on our results, the functional characterization of the *B*. *cusia* genes mentioned above should be further investigated.

## Results

### Quantitative analysis with HPLC

**Fig 1:**
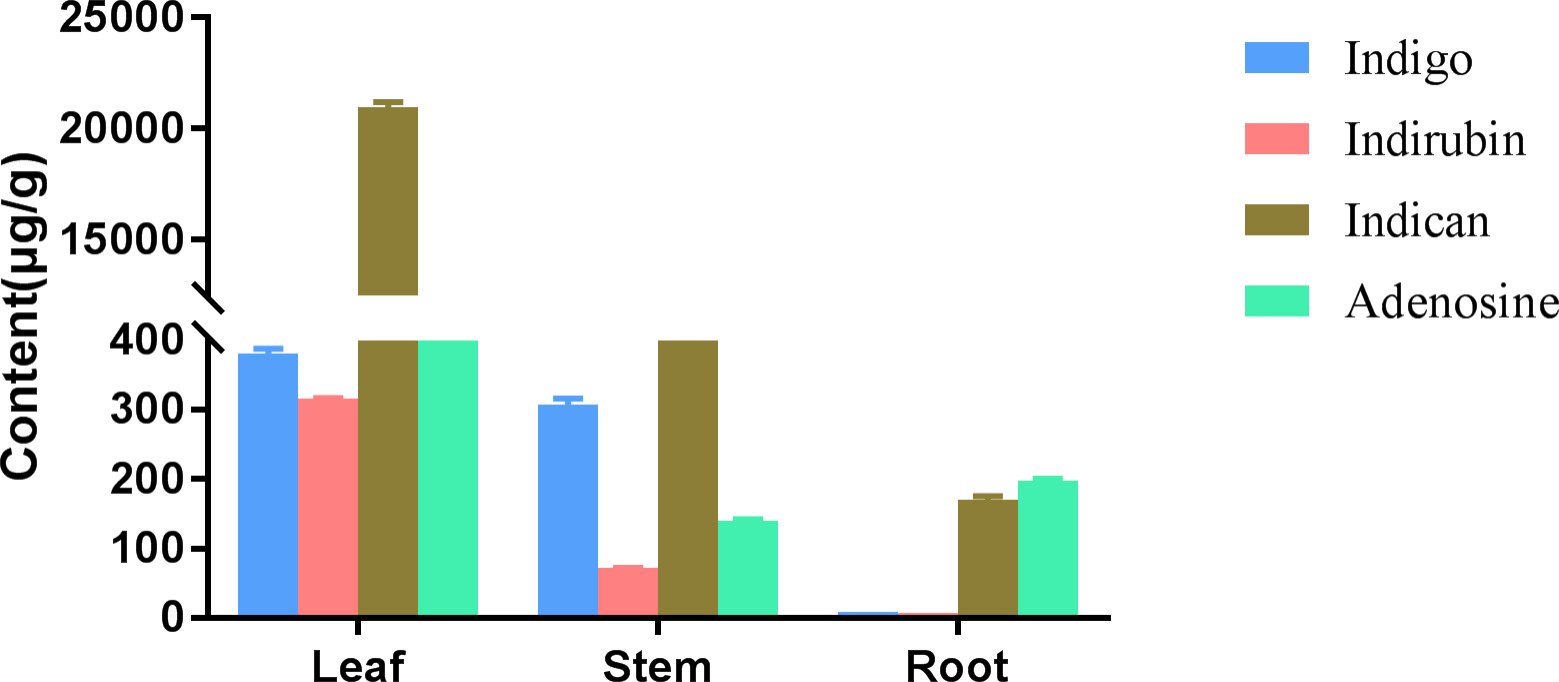
Contents of four ingredients in leaves, stems and roots of *B*. *cusia*. The experiment was repeated three times. The amounts of indigo, indirubin, indican and adenosine in leaf tissue (380.66μg/g, 315.15 μg/g, 20,978.26 μg/g and 1224.12 μg/g, respectively) were higher than those in the stem tissue (306.36 μg/g, 71.71μg/g, 3056.78 μg/g and 139.45 μg/g, respectively, *p* = 0.000), the amounts of indigo, indirubin and indican in stem tissue were higher than those in the root tissue (9.31 μg/g, 7.82 μg/g and 170.45 μg/g, respectively, *p* = 0.000), and the amount of adenosine in stem tissue was lower than that in root tissue (197.48 μg/g), but the difference was not significant (*p* = 0.350).

The calibration curves of indigo, indirubin, adenosine and indican were formulated with five different concentration references. The regression equation was Y = aX + b, in which X and Y are the concentration and peak area, respectively. The regression equations and correlation coefficients (r^2^) of indigo, indirubin, adenosine and indican were as follows: Y = 25561X + 283.01(lineat range from 0.188 to 23.5μg∙ml^-1^), r^2^ = 1.0000 for indigo; Y = 39667X - 1296.9(linear range from 0.092 to 11.5 μg∙ml^-1^), r^2^ = 1.0000 for indirubin, Y = 15817X + 28642(linear range from 1.1 to 275μg∙ml^-1^), r^2^ = 0.9993 for adenosine, and Y = 6020.5X - 5001(linear range from 1.08 to 270 μg∙ml^-1^), r^2^ = 1.0000 for indican. The relative quantity of four ingredients in the leaf, stem and root samples (μg/g) was calculated using the above equations (Fig 1).

### RNA sequencing and de novo assembly

After total RNA was extracted, mRNA isolated from leaves, stems and roots of *B*. *cusia* (three-year-old plants cultivated) was enriched by oligo (dT) beads in order to acquire a general set of *B*. *cusia* transcripts. After removing reads containing adaptors or low-quality paired-end reads, clean and high-quality reads were analysed. A total of 140,240,688 high quality reads were obtained for root, 137,216,248 for leaf and 122,837,394 for stem. Contig and unigene de novo assembly was carried out with the short read assembling program Trinity using the high-quality reads. 63,327,716 bases, 51,381 unigenes and 1,932 bp N50, and average length 1,232 bp were assembled. The GC percentage was 42.4176.

### Annotation of the unigenes

**Fig 2:**
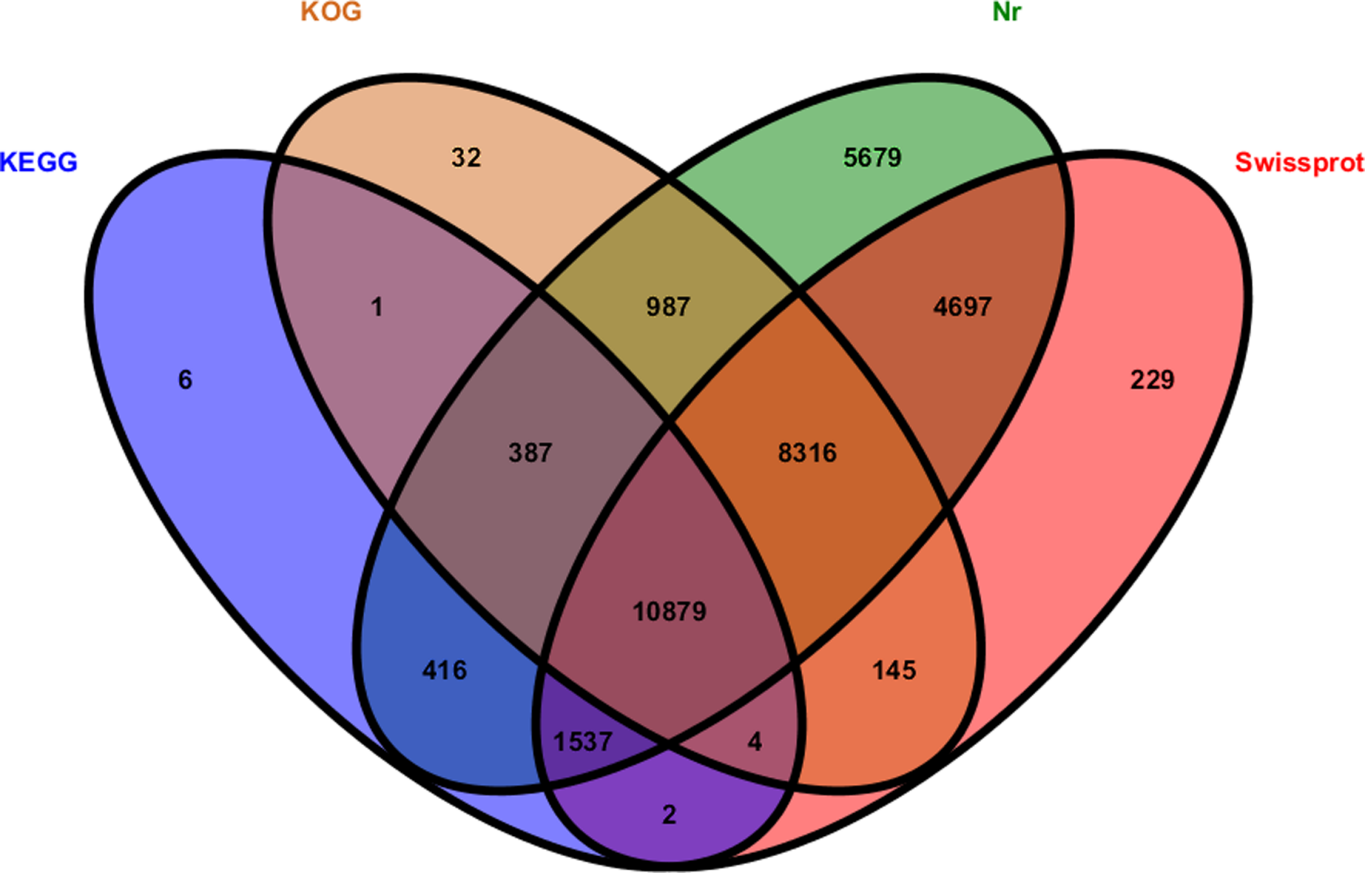
Venn diagram of unigene number annotated by BLASTx with the Nr, SwissProt, KOG and KEGG databases.

51,381 unigenes were widely searched against various databases in order to determine the potential functions of the assembled unigenes. To annotate the unigenes, we used the BLASTx program with an E-value threshold of 10^−5^ and a *k*-mer of 25 with the NCBI non-redundant protein (Nr) database, the Swiss-Prot protein database, the Kyoto Encyclopaedia of Genes and Genomes (KEGG) database, and the COG/KOG database. Unigene functional annotations were obtained according to the best alignment results. The results showed that 32,898, 25,809, 20,751 and 13,232 unigenes matched with the Nr, SwissProt, KOG and KEGG databases, respectively. 33,317 unigenes matched with one of the above databases (Fig 2).

**Fig 3:**
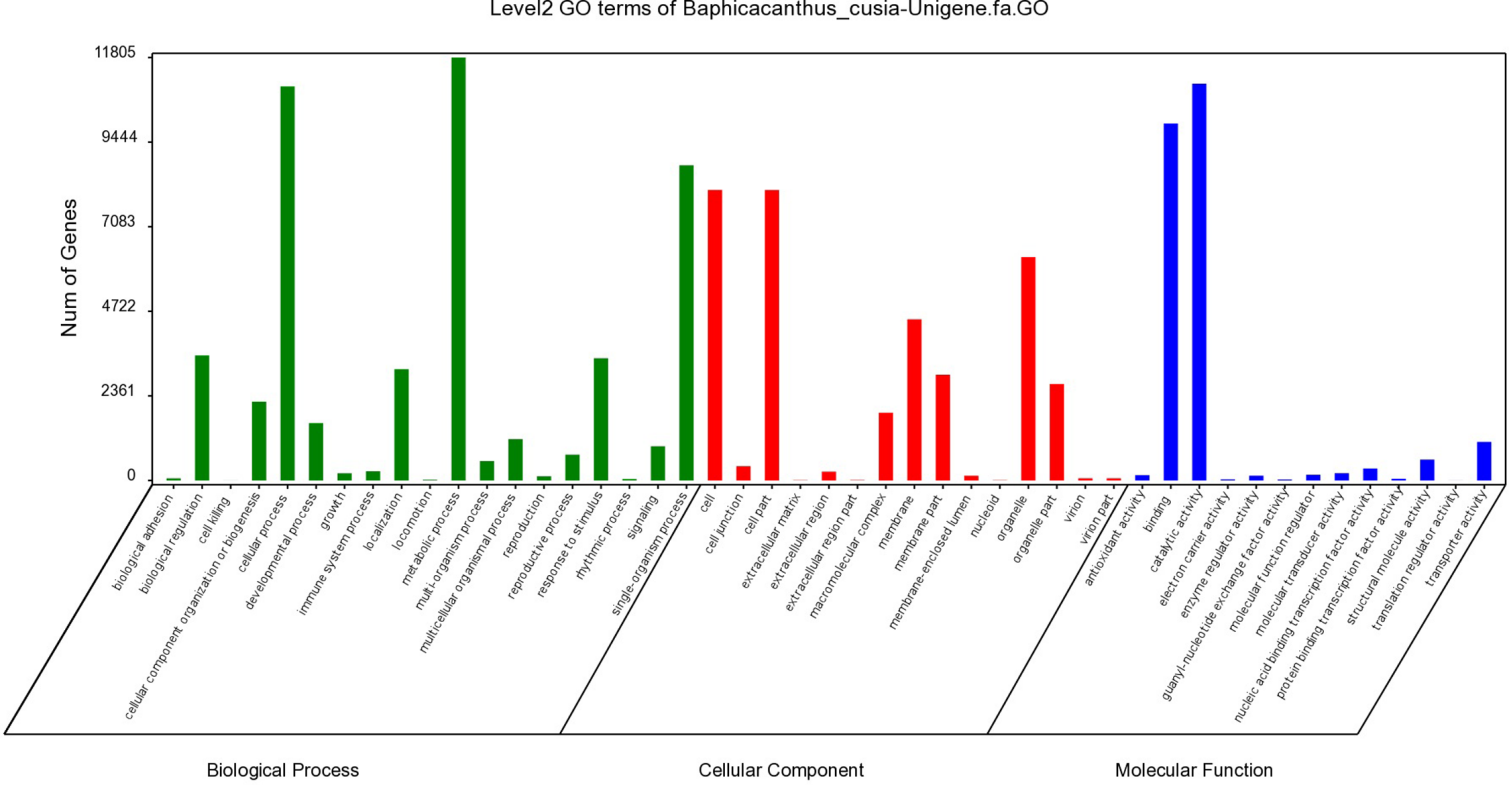
Histogram of level 2 GO terms of the *B*. *cusia* unigenes.

GO functional annotations of *B*.*cusia* unigenes were obtained from Nr annotation results. Based on their GO terms, the unigenes could be categorized into three main GO groups and 47 functional sub-groups (Fig 3). The three most frequent GO terms in biological process were metabolic processes (11,803 unigenes), cellular processes (10,998 unigenes), and single-organism processes (8,797 unigenes). The three most frequent GO terms in cellular components were cells (8,107 unigenes), cell parts (8,106 unigenes), and organelles (6,236 unigenes). The three most frequent GO terms in molecular function groups were catalytic activity (11,075 unigenes), binding (9,963 unigenes), and transporter activity (1,076 unigenes).

**Fig 4:**
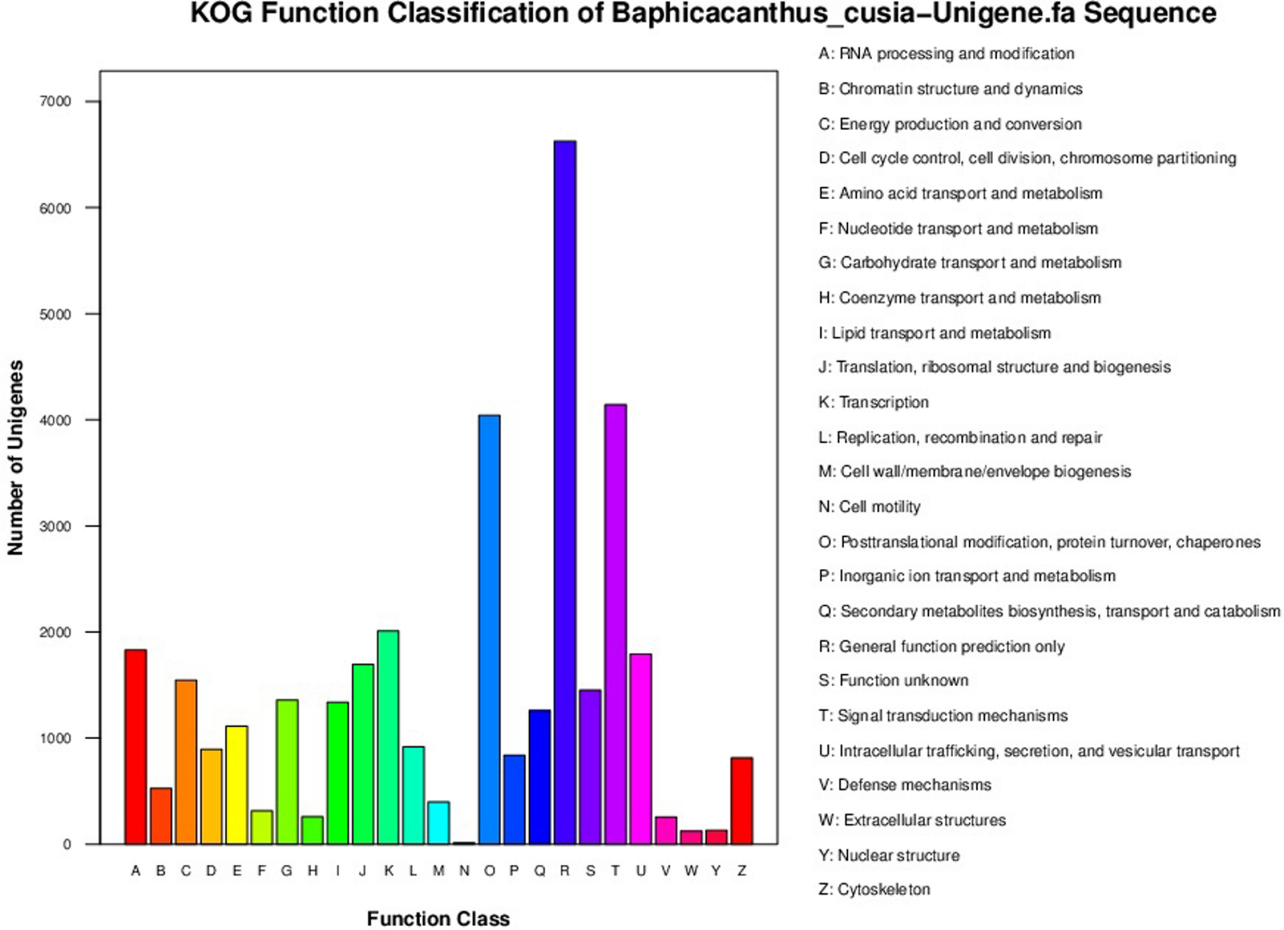
Histogram of KOG function classification of *B*. *cusia* unigenes.

35,671 of the total 51,381 *B*. *cusia* unigenes from analysis of EuKaryotic Orthologous Groups (KOG) could be classified into 25 clusters. Among the classified unigenes, the most part of the cluster was “general function prediction” (6,624; 18.57%), which was followed by clusters of “signal transduction mechanisms” (4,143; 11.61%), “posttranslational modification, protein turnover, chaperones” (4,042; 11.33%), “transcription” (2,009; 5.43%), “RNA processing and modification” (1,830; 5.13%) and intracellular trafficking, secretion, and vesicular transport (1,791; 5.02%). The cluster of the least number was “cell motility” (14; 0.04%) (Fig 4).

### KEGG pathway mapping

**Fig 5:**
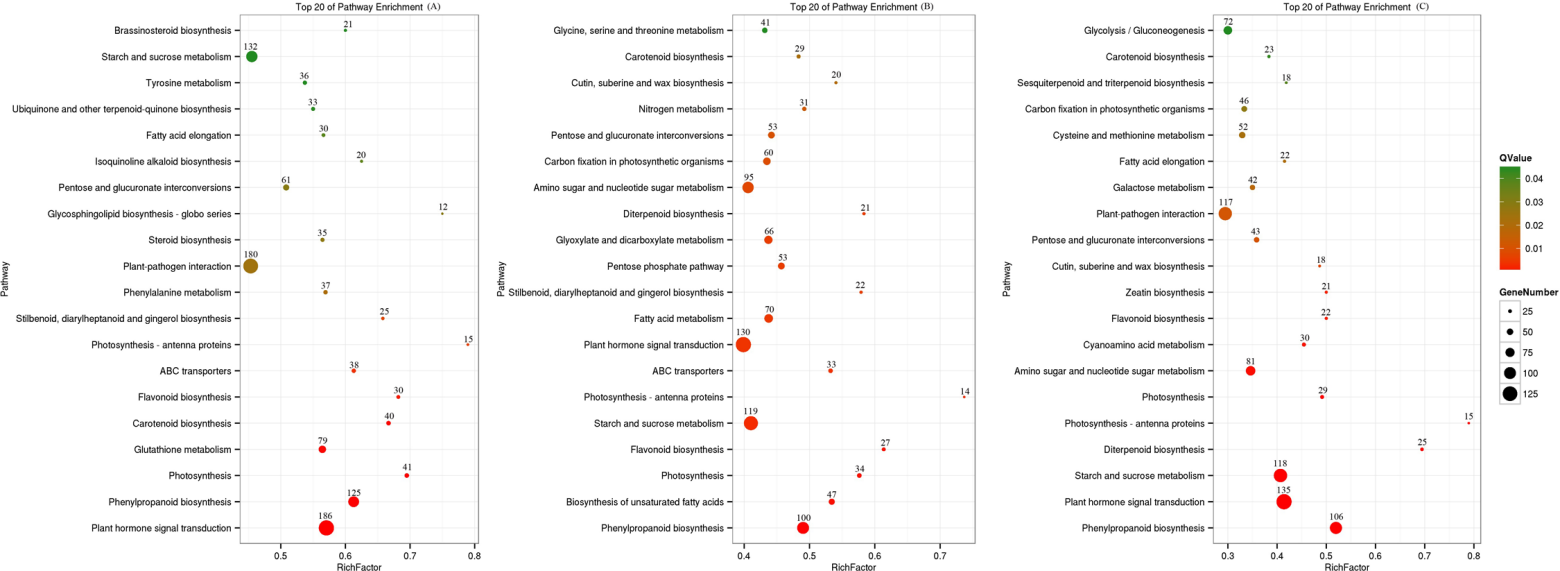
Top 20 pathways enriched among various tissues of *B*. *cusia*. (A) The top 20 pathways enriched between root and leaf; (B) the top 20 pathways enriched between root and stem; (C) the top 20 pathways enriched between stem and leaf. The top 20 pathways enriched from top to bottom are arranged by decreasing Q-value. Data over the dots show the actual gene number.

To better understand the biological functions and pathways represented by the unigenes assembled in this study, 7,350 unigenes were classified into 127 pathways. The pathways included metabolism, genetic information processing, cellular processes, environmental information processing, and organismal systems. The top 20 pathways, which have predominant sequences, are indicated in Fig 5 (S1–S3 Tables), and the most represented pathways which were enriched in the DEG sets compared to the total transcriptome were plant hormone signal transduction and phenylpropanoid biosynthesis. Rich factors were 0.57, 0.40, 0.41 for plant hormone signal transduction (Q-value 3.36E-10, 3.92E-03, 7.03E-13) and 0.61, 0.49, 0.52 for phenylpropanoid biosynthesis (Q-value 1.20E-9, 3.66E-06, 3.70E-18) between root and leaf, root and stem, and stem and leaf, respectively. The main purpose of cultivating *B*. *cusia* has been to obtain the Indigo Naturalis, which has active ingredients of indole alkaloids and their derivatives. In this study, the annotated results suggest 63 unigenes related to nitrogen metabolism (S4 Table) and 58 unigenes connected to phenylalanine, tyrosine and tryptophan biosynthesis (biosynthesis of indole and its derivatives) (S5 Table).

### Identification of simple sequence repeats

**Fig 6:**
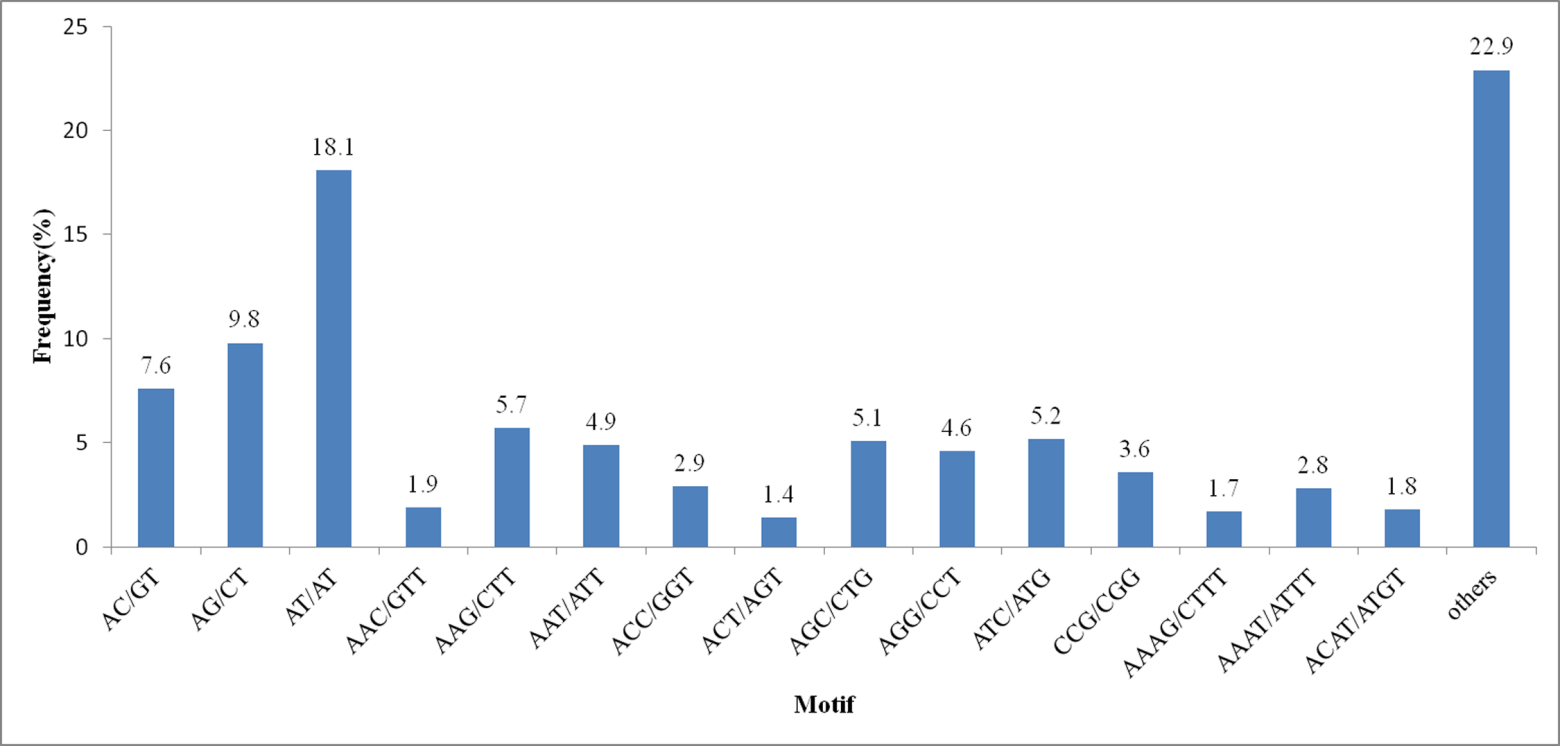
Distribution of various types of SSRs identified in *B*. *cusia* unigenes.

To find SSR markers, MISA software was employed for microsatellite mining in the whole *B*. *cusia* transcriptome. Based on the MISA results, three pairs of PCR primers have been designed by Primer 3 in the flanking regions of SSRs (S6 Table). Within the 51,381 examined unigenes, 6,782 unigenes were identified containing 8,471 SSR markers, and 1,350 unigenes contained more than one SSR. Tri-nucleotides were the most abundant SSR (3,046; 35.96%), followed by di-nucleotides (3,021; 35.66%), tetra-nucleotides (1,065; 12.57%), hexa-nucleotides (861; 10.16%), and penta-nucleotides (478; 5.64%) were the least frequent SSR (S7 Table). Among the confirmed SSRs, 547 showed a compound formation. There were 16 types of SSR identified in *B*. *cusia* (Fig 6). Until now, there have been no publicly available SSRs in *B*. *cusia*, and thus the SSRs identified in this study will be extremely helpful for research on genotyping applications in *B*. *cusia*.

### Analysis of differentially expressed genes

There were 13,836 genes expressed differentially between roots and stems. Among these differentially expressed unigenes, 6,633 were up-regulated and 7,203 were down-regulated in roots compared to stems. There were 20,083 unigenes differentially expressed between roots and leaves; among these differentially expressed unigenes, 5,383 were up-regulated and 14,700 were down-regulated in roots compared to leaves. There were 11,532 unigenes differentially expressed between stems and leaves; among these differentially expressed unigenes, 2,491 were up-regulated and 9,041 were down-regulated in stems compared to leaves.

In light of the putative biosynthetic pathways of indole derivatives in *Isatis indigotica* [34], analyse of the differentially expressed genes among various tissues were carried out for biosynthesis of indican, cytochrome P450 (CYP450) [34], UDP-glycosyltransferase (UGT) [34], glucosidase [45] and tryptophan synthase; these were chosen to be analysed after annotation.

**Fig 7:**
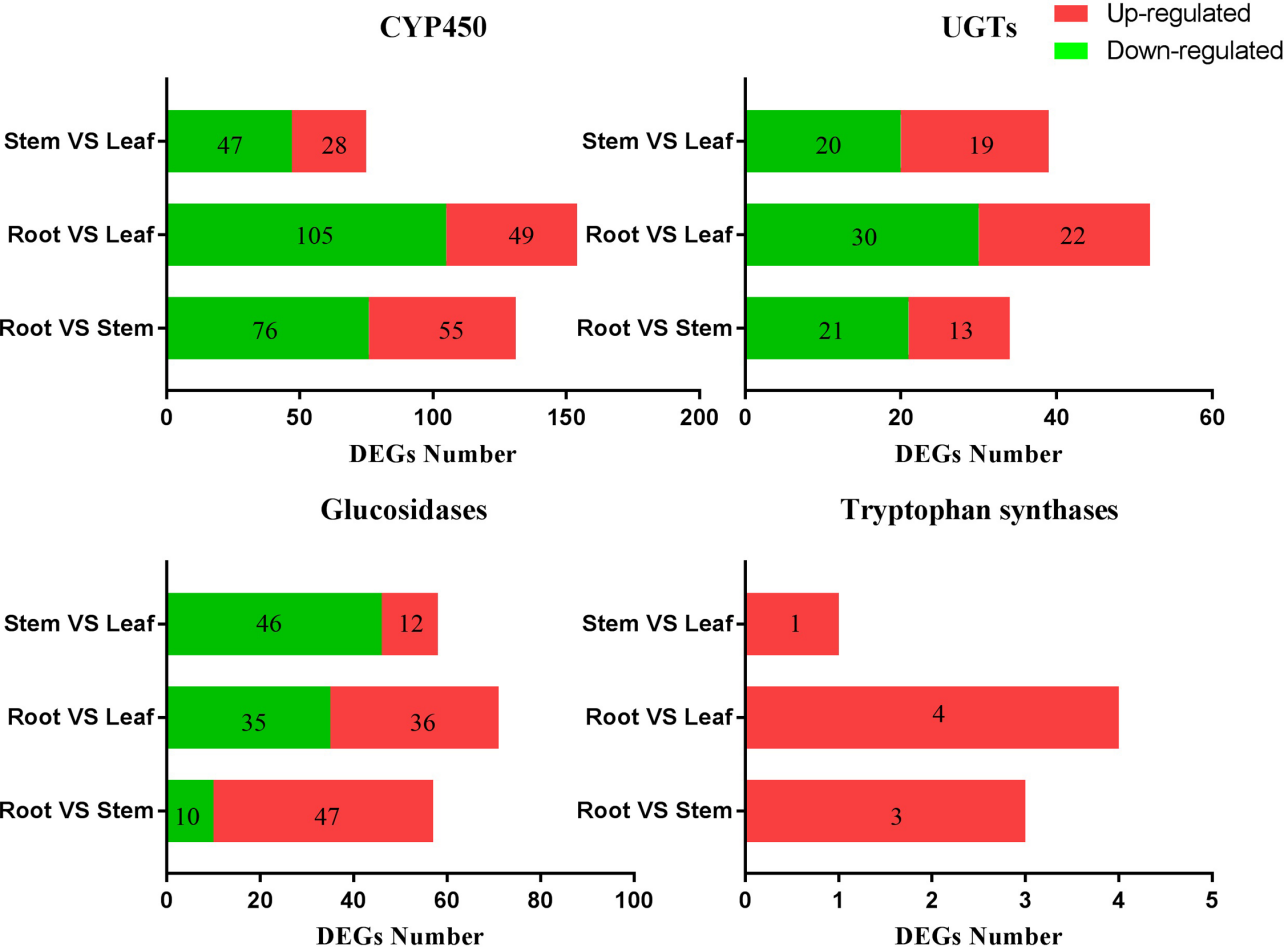
Annotated CYP450, UGT, glucosidase, and tryptophan synthase DEG numbers among various tissues of *B*. *cusia*. The data in green bars show the down-regulated gene number and the data in red bars show the up-regulated gene number.

There were 121 CYP450, 34 UGT, 57 glucosidase, and three tryptophan synthase DEGs that were differentially expressed between roots and stems (S8 Table). There were 154 CYP450, 52 UGTs, 71 glucosidase, four tryptophan synthase DEGs between roots and leaves (S9 Table). There were 75 CYP450, 39 UGT, 58 glucosidase, and one tryptophan synthase DEG between stems and leaves (S10 Table and Fig 7); the DEGs of stems were more similar to leaves than those of roots.

### Validation of DEG data by qRT-PCR

**Fig 8:**
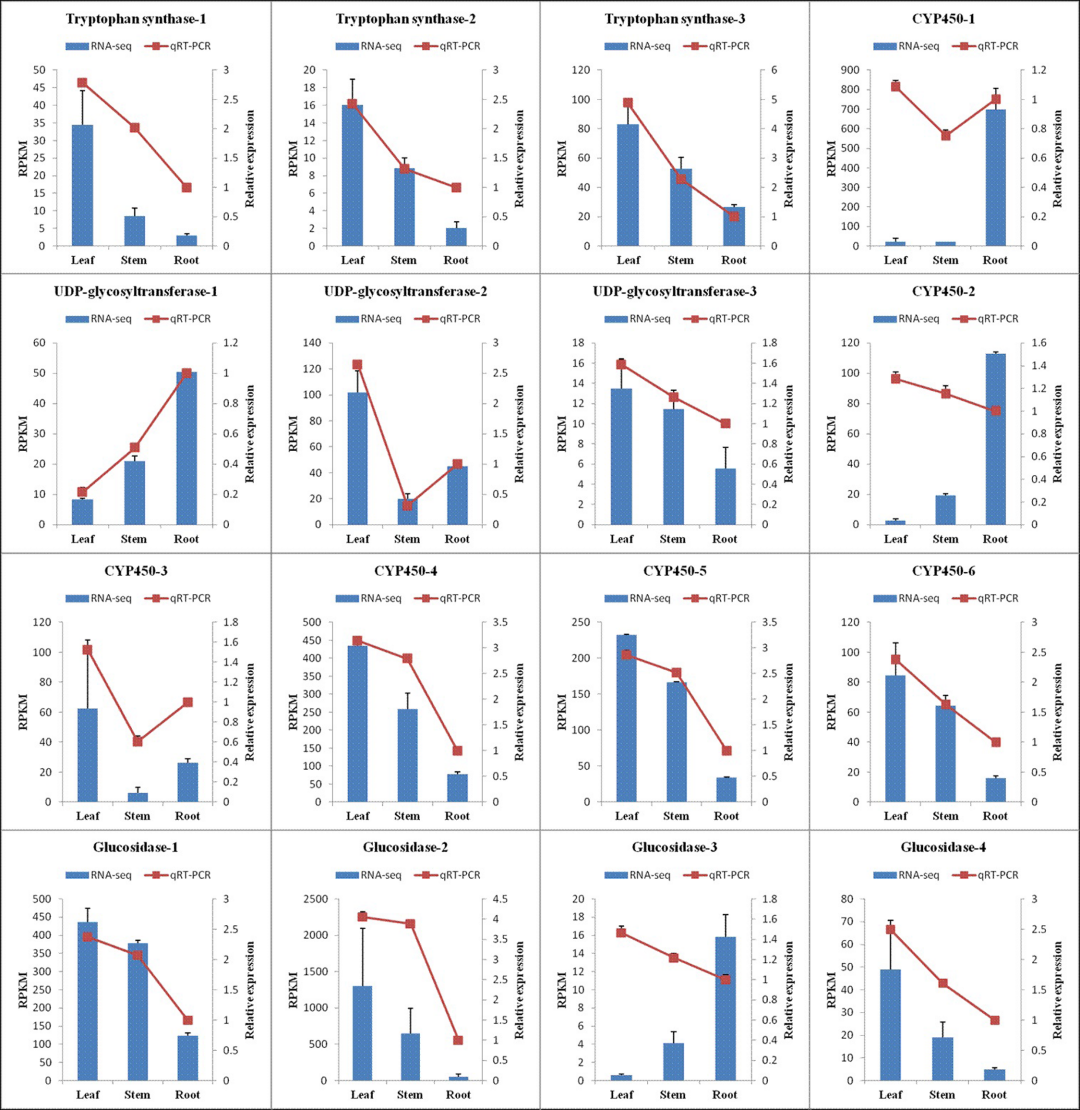
qRT-PCR validation of DEGs identified by RNA-Seq. Sixteen DEGs involved in the biosynthesis and metabolism of indican were randomly selected for qRT-PCR confirmation. The blue bars and red lines represent RNA-Seq RPKM data and qRT-PCR relative expression levels, respectively. The beta-actin gene was used as an internal control, and every experiment was repeated with three biological samples. All the values shown are the means ± SE.

We performed qRT-PCR on 16 representative unigenes related to the biosynthesis and metabolism of indican to verify the DEGs acquired by Illumina RNA-Seq. We found that while three DEGs (two CYP450 unigenes and one glucosidase unigene) did not show consistent expression between qRT-PCR and RNA-Seq data sets, most of the DEGs from qRT-PCR exhibited similariry to RNA-Seq samples using qRT-PCR (Fig 8 and S11 Table). Compared with leaves, tryptophan synthase expression was down-regulated in roots and stems, which agreed with the results of quantitative determination of indican among various tissues of *B*. *cusia*.

## Discussion

In this study, the amounts of four active ingredients, such as indigo, indirubin, indican and adenosine, were found to differ substantially in root, stem and leaf tissues from *B*. *cusia*. The amounts of indigo, indirubin, indican and adenosine in leaf tissue were 380.66μg/g, 315.15 μg/g, 20,978.26 μg/g and 1224.12 μg/g; these components in stem tissue were 306.36 μg/g, 71.71μg/g, 3056.78 μg/g and 139.45 μg/g, in root tissue were 9.31 μg/g, 7.82 μg/g, 170.45 μg/g and 197.48 μg/g respectively. The amounts of indigo and indirubin in leaf tissue were higher than those in the root tissue (*p*<0.01), which was consistent with a previous report [20]. This is the first study of the amounts of adenosine and indican simultaneously in the leaf and root tissues of *B*.*cusia*. Our work provides novel information with respect to the mechanism of different tissues of *B*. *cusia* used as natural medicine, since this may be related to the distribution of indican and the metabolites indigo and indirubin.

A total of 63 unigenes related to nitrogen metabolism pathways and 58 unigenes involved in the biosynthesis pathway of indole and its derivatives were found. Further studies of these unigenes will help to understand regulation of secondary metabolism in *B*. *cusia*. In addition, 154 CYP450, 52 UGT, 71 glucosidase and four tryptophan synthase genes were differentially expressed between leaves and roots, 75 CYP450, 39 UGT, 58 glucosidase and one tryptophan synthase gene were differentially expressed between leaves and stems, 121 CYP450, 34 UGT, 57 glucosidase and three tryptophan synthase gened were differentially expressed between stems and roots. Tryptophan synthase DEGs may be the candidate genes contributing to the production of indigo and indirubin in *B*. *cusia* leaves.

In addition, there were 125 DEGs involved in phenylpropanoid biosynthesis, 30 in flavonoid biosynthesis, and 35 in the terpenoid backbone biosynthesis pathway between roots and leaves; there were 106 DEGs involved in phenylpropanoid biosynthesis, 22 in flavonoid biosynthesis, and 23 in the terpenoid backbone biosynthesis pathway between stems and leaves, and there were 100 DEGs involved in phenylpropanoid biosynthesis, 27 in flavonoid biosynthesis, and 25 in the terpenoid backbone biosynthesis pathway between roots and stems. The investigation of these unigenes may shed light on the molecular mechanisms of secondary metabolism in *B*. *cusia*.

In summary, there were 51,381 unigenes from the leaf, stem and root transcriptomes of *B*. *cusia* using RNA sequencing in this study, but only 64.84% of the unigenes could be annotated by the Nr, SwissProt, KOG or KEGG databases. There were a great many unannotated unigenes and their biological pathways remain to be identified. Util now, this has been the first report of the transcriptome of *B*. *cusia*; these unigenes could be useful for further investigating genes related to abiotic stress in *B*. *cusia*, accelerating the research on the molecular mechanisms of secondary metabolism in stress responses and differential metabolic patterns of compounds such as indican, indigo and indirubin in different components of *B*. *cusia*.

## Materials and methods

### Ethics statement

All necessary permits were acquired for these studies. Samples were collected from our medicinal garden for scientific research at the Fujian Agricultural and Forestry University. We confirm that endangered or protected species were not contained in the species collections.

### Plant materials and RNA sequencing

Three batches of root, stem and leaf samples were collected from three-year-old plants cultivated in our medicinal garden, frozen in liquid nitrogen for 15 min immediately, and then stored at −80°C refrigerator for future use. The EASYspin plant RNA kit (Aidlab Biotechnologies Co.Ltd, Beijing, China) was used to isolate total RNA, and contaminated DNA was removed by DNase I. An Agilent Bioanalyzer 2100 (Agilent Technologies) was used to analyse the quality and integrity of RNA. cDNA fragments were purified using the QiaQuick PCR extraction kit, end repaired, poly (A) was added, and then the fragments were ligated to Illumina sequencing adapters. Sequenced were obtained using the Illumina HiSeqTM 4000 by Gene Denovo Biotechnology Co. (Guangzhou, China).

### Analysis with high performance liquid chromatography

Reverse-phase high performance liquid chromatography was performed on an LC-20AT HPLC system (Shimadzu, Japan), which included a LC-20AT pump, DGU-20A degasser, SIL-20A autosampler, SPD-20A detector and CBM-20A central controller, using a C_18_ reverse phase chromatographic column (Inertsil ODS-SP, 4.6×250 mm, 5 μm) at 25°C, with wavelengths monitored at 292 nm for indigo and indirubin and 260 nm for adenosine and indican. The mobile phase included methanol and water, with a volume ratio of 70: 30 for both indigo and indirubin and volume ratio of 15: 85 for adenosine and indican with a mobile phase flow rate of 1.0 ml/min. To prepare the sample and reference solutions, three batches of leaf, stem and root samples were extracted twice by a Soxhlet extractor. Indigo and indirubin mixed reference solutions, in triplicate, were dissolved in N, N-dimethyl formamide, and adenosine and indican mixed reference solutions, in triplicate, were dissolved in 30% methanol. After filtration through a 0.45 μm filter membrane, 10 μl of each sample and reference solution was injected in duplicate. The chromatographic peak retention times of indigo and indirubin and adenosine and indican were approximately 15 min and 20 min, respectively.

### Transcriptome *de novo* assembly and bioinformatic analysis

Raw reads generated from HiSeq 4000 were further filtered to remove adapters and low-quality sequences. The *de novo* assembling program Trinity consists of three components: Inchworm, Chrysalis and Butterfly; *de novo* assembly was generated with the clean reads. In brief, we first removed the wrong k-mers, constructed a k-mer library from the clean reads and then generated a collection of linear contigs by a greedy extension based on (k-1)-mer overlaps. Next, we clustered related contigs into components and built a de Bruijn graph for each cluster of related contigs. Finally, we simplified the de Bruijn graph, then unlinked the de Bruijn graph using real reads and reconstructed the transcript sequences.

All assembled unigenes were annotated by BLASTx with the NCBI non-redundant (Nr) protein database, Swiss-Prot, the Kyoto Encyclopaedia of Genes and Genomes (KEGG) database and EuKaryotic Orthologous Groups (KOG). Sequences with BLASTx hits were determined according to Gene Ontology (GO) terms by Blast2GO software. The functional classification of unigenes was performed by WEGO software. SSRs were scanned by MISA (MIcroSAtellite identification tool) software (http://pgrc.ipk-gatersleben.de/misa/) with the default software parameter settings. To study the differentially expressed genes, the log2-converted values of each contig were subjected to a Benjamini-Hochberg program. The differential expression of genes was counted with the FDR value and FDR fold change (fc). If the FDR value < 0.05 and |log2fc| > 1, then the gene was considered differential. Unigene expression was counted and normalized to RPKM (reads per kb per million reads) [46]. The formula is as follows: RPKM = (1000000 * C)/(N * L / 1000), given RPKM (A) as the expression of Unigene A, C is the number of reads mapped to Unigene A uniquely, N is the total number of reads mapped to all unigenes uniquely, L is the base number length of Unigene A.

Pathway enrichment analysis identified significantly enriched metabolic pathways or signal transduction pathways in DEGs compared to the whole genome background. The formula to calculate the *P*-value is:

$$\text{P=1-}\sum_{\text{i=0}}^{\text{m-1}}\frac{\left(\begin{array}{c} \text{M}\\ \text{i} \end{array}\right)\left(\begin{array}{c} \text{N-M}\\ \text{n-i} \end{array}\right)}{\begin{array}{c} \left(\begin{array}{c} \text{N}\\ \text{n} \end{array}\right) \end{array}}$$ (1)

Here, N is the number of all genes with KEGG annotation, n is the number of DEGs in N, M is the number of all genes annotated to specific pathways, and m is the number of DEGs in M. The calculated *p*-value was determined through FDR correction, taking FDR ≤ 0.05 as a threshold. Pathways meeting this condition were defined as significantly enriched pathways in DEGs. Rich factor refers to the ratio of the number of genes located in the pathway entry in the differentially expressed genes to the total number of genes located in the pathway entry in all genes. Q-value is the *P*-value after multi-hypothese test correction. The value range is 0 to 1.

## Supporting information

S1 Table Top 20 pathways enriched between root and leaf. (XLSX)

S2 Table Top 20 pathways enriched between root and stem. (XLSX)

S3 Table Top 20 pathways enriched between stem and leaf. (XLSX)

S4 Table KEGG pathway mapping and unigenes involved in nitrogen metabolism. (XLSX)

S5 Table KEGG pathway mapping and unigenes involved in phenylalanine, tyrosine and tryptophan biosynthesis. (XLSX)

S6 Table Primers for SSRs identified in the *B*. *cusia* unigenes. (TXT)

S7 Table Statistics of the SSRs identified in the *B*. *cusia* unigenes. (XLSX)

S8 Table Cytochrome P450, UDP-glycosyltransferase, glucosidase, tryptophan synthase DEGs between root and stem tissues of *B*. *cusia* unigenes. (XLSX)

S9 Table Cytochrome P450, UDP-glycosyltransferase, glucosidase, tryptophan synthase DEGs between root and leaf tissues of *B*. *cusia* unigenes. (XLSX)

S10 Table Cytochrome P450, UDP-glycosyltransferase, glucosidase, tryptophan synthase DEGs between stem and leaf tissues of *B*. *cusia* unigenes. (XLSX)

S11 Table Nucleotide sequences of primers specific to indican metabolic related genes used for qRT-PCR. (XLSX)
